# Noradrenergic activation of the basolateral amygdala enhances object recognition memory and induces chromatin remodeling in the insular cortex

**DOI:** 10.3389/fnbeh.2015.00108

**Published:** 2015-04-28

**Authors:** Hassiba Beldjoud, Areg Barsegyan, Benno Roozendaal

**Affiliations:** ^1^Department of Cognitive Neuroscience, Radboud University Medical CenterNijmegen, Netherlands; ^2^Donders Institute for Brain, Cognition and Behaviour, Radboud University NijmegenNijmegen, Netherlands

**Keywords:** norepinephrine, emotional arousal, memory consolidation, epigenetics, histone acetylation

## Abstract

It is well established that arousal-induced memory enhancement requires noradrenergic activation of the basolateral complex of the amygdala (BLA) and modulatory influences on information storage processes in its many target regions. While this concept is well accepted, the molecular basis of such BLA effects on neural plasticity changes within other brain regions remains to be elucidated. The present study investigated whether noradrenergic activation of the BLA after object recognition training induces chromatin remodeling through histone post-translational modifications in the insular cortex (IC), a brain region that is importantly involved in object recognition memory. Male Sprague—Dawley rats were trained on an object recognition task, followed immediately by bilateral microinfusions of norepinephrine (1.0 μg) or saline administered into the BLA. Saline-treated control rats exhibited poor 24-h retention, whereas norepinephrine treatment induced robust 24-h object recognition memory. Most importantly, this memory-enhancing dose of norepinephrine induced a global reduction in the acetylation levels of histone H3 at lysine 14, H2B and H4 in the IC 1 h later, whereas it had no effect on the phosphorylation of histone H3 at serine 10 or tri-methylation of histone H3 at lysine 27. Norepinephrine administered into the BLA of non-trained control rats did not induce any changes in the histone marks investigated in this study. These findings indicate that noradrenergic activation of the BLA induces training-specific effects on chromatin remodeling mechanisms, and presumably gene transcription, in its target regions, which may contribute to the understanding of the molecular mechanisms of stress and emotional arousal effects on memory consolidation.

## Introduction

Enhanced memory for emotionally arousing events is a well-recognized phenomenon, which has obvious adaptive value in evolutionary terms, as it is vital to remember both dangerous and favorable situations (Roozendaal and McGaugh, 2011). Extensive evidence indicates that noradrenergic activation of the basolateral complex of the amygdala (BLA) is critically involved in mediating emotional arousal effects on memory enhancement by influencing synaptic plasticity and information storage processes in other brain regions (Introini-Collison et al., 1991; Ferry et al., 1999; Hatfield and McGaugh, 1999; Roozendaal et al., 2002, 2009; LaLumiere et al., 2003; Huff et al., 2005; Barsegyan et al., 2014). Noradrenergic activation of the BLA also enhances the consolidation of low-arousing object recognition memory (Roozendaal et al., 2008), a naturalistic task based on the spontaneous tendency of rodents to explore a novel object more than a familiar one (Ennaceur and Delacour, 1988). Memory for object recognition training is known to depend on synaptic plasticity changes within the perirhinal cortex (Albasser et al., 2009; Barker and Warburton, 2011) and insular cortex (IC; Bermúdez-Rattoni et al., 2005; Balderas et al., 2008; Roozendaal et al., 2010; Bermúdez-Rattoni, 2014). For example, long-term memory of an object, but not of the location of the object, is impaired when the protein-synthesis blocker anisomycin is applied into either the perirhinal cortex or IC, whereas short-term memory is not affected (Balderas et al., 2008). Considerable evidence indicates that the IC, which has traditionally been investigated mostly with respect to its involvement in taste memory (Berman et al., 2000; Bermúdez-Rattoni, 2004; Shema et al., 2007; Núñez-Jaramillo et al., 2010; Stehberg et al., 2011), is an important node of the rodent brain network involved in emotional regulation of learning and memory (Bermúdez-Rattoni and McGaugh, 1991; Bermúdez-Rattoni et al., 1991; Nerad et al., 1996; Fornari et al., 2012b). The IC is also densely interconnected with the BLA (McDonald and Jackson, 1987; Paré et al., 1995; Shi and Cassell, 1998) and several studies indicated functional interactions between these two brain regions (Escobar et al., 1998; Rodríguez-Durán et al., 2011; Moraga-Amaro and Stehberg, 2012). Critically, the finding that noradrenergic blockade of the BLA prevents the effect of drug administration into the IC on conditioned taste aversion as well as inhibitory avoidance memory (Miranda and McGaugh, 2004), provides important support for the view that noradrenergic activity of the BLA regulates neural plasticity and memory consolidation processes within this brain region.

Whereas the behavioral consequences of noradrenergic activity of the BLA on memory consolidation are well established, the molecular mechanism(s) underlying this BLA influence on information storage processes in efferent brain regions are yet to be determined. During the last decade new investigations concerning the mechanism of gene expression have shed light on different forms of epigenetic modifications, i.e., histone post-translational modifications (PTMs), DNA methylation and non-coding RNAs, that are involved in learning and memory (Levenson et al., 2004; Miller et al., 2008, 2010; Stefanko et al., 2009; Gupta et al., 2010; Roozendaal et al., 2010; Reolon et al., 2011). An impressive body of literature indicates that the chromatin state through histone PTMs, such as acetylation, phosphorylation or methylation of histone tails, must be altered to allow for changes in gene expression related to memory consolidation (Levenson et al., 2004; Chwang et al., 2006; Koshibu et al., 2009; Stefanko et al., 2009; Gupta et al., 2010; Roozendaal et al., 2010; Haettig et al., 2011; Kye et al., 2011; Gräff et al., 2012; Griggs et al., 2013). In a prior study, we reported that inducing a histone hyperacetylated state within the IC with local posttraining infusions of the histone deacetylase (HDAC) inhibitor sodium butyrate (NaB) enhanced memory of object recognition training (Roozendaal et al., 2010). Here, we investigated whether a memory-enhancing dose of norepinephrine administered into the BLA after object recognition training triggers chromatin modifications within the IC. We examined different histone PTM marks that have been reported to be involved in learning and memory and/or stress adaptation such as acetylation of histone H3 at lysine 14 (acH3K14), acetylation of histone H2B (acH2B), acetylation of histone H4 (acH4), phosphorylation of histone H3 at serine 10 (pH3S10) and tri-methylation of histone H3 at lysine 27 (3meH3K27) (Chwang et al., 2006; Fischer et al., 2007; Hunter et al., 2009; Koshibu et al., 2009, 2011; Bousiges et al., 2010; Gräff et al., 2012). To determine whether the norepinephrine effect on histone PTMs depends on the object recognition training experience, changes in these histone marks were also assessed after norepinephrine administration into the BLA of non-trained control rats. Furthermore, as some findings indicated an important role for extracellular signal-regulated kinase 1/2 (ERK1/2) protein in regulating histone PTMs in memory formation (Levenson et al., 2004; Chwang et al., 2006; Chandramohan et al., 2008; Gutièrrez-Mecinas et al., 2011; Mifsud et al., 2011), we also investigated whether noradrenergic activation of the BLA after object recognition training changes the phosphorylation status of ERK1/2 in the IC.

## Materials and Methods

### Subjects

Male adult Sprague-Dawley rats (280–320 g at time of surgery) from Charles River Breeding Laboratories (Kisslegg, Germany) were housed individually in a temperature-controlled (22°C) vivarium room and maintained on a 12-h:12-h light:dark cycle (lights on: 7:00–19:00 h) with *ad libitum* access to food and water. Training and testing were performed during the light phase of the cycle between 10:00 and 15:00 h. All experimental procedures were in compliance with the European Communities Council Directive on the use of laboratory animals of November 24, 1986 (86/609/EEC) and approved by the Institutional Animal Care and Use Committees of the University of Groningen and Radboud University Nijmegen, Netherlands.

### Surgery

Rats, adapted to the vivarium for 1 week, were anesthetized with a subcutaneous injection of ketamine (37.5 mg/kg of body weight; Alfasan) and dexmedetomidine (0.25 mg/kg; Orion) and received the non-steroidal analgesic carprofen (4 mg/kg; Pfizer). Oxygen (35%) mixed with ambient air was administered during surgery such that blood oxygenation levels would not drop below 90% (Fornari et al., 2012a). The rats were positioned in a stereotaxic frame (Kopf Instruments, Tujunga, CA), and two stainless-steel guide cannulae (15 mm; 23 gauge; Component Supply Co/SKU Solutions, Fort Meade, FL) were implanted bilaterally with the cannula tips 2.0 mm above the BLA. The coordinates were based on the atlas of Paxinos and Watson (2007): anteroposterior (AP), −2.8 mm from Bregma; mediolateral (ML), ±5.0 mm from the midline; dorsoventral (DV), −6.5 mm from skull surface; incisor bar: −3.3 mm from interaural. The cannulae were affixed to the skull with two anchoring screws and dental cement. Stylets (15-mm-long 00 insect dissection pins) were inserted into each cannula to maintain patency. After surgery, the rats were administered atipamezole hydrochloride (0.25 mg/kg sc; Orion) to reverse anesthesia and were subsequently injected with 3 ml of sterile saline to facilitate clearance of drugs and prevent dehydration. The rats were allowed to recover for a minimum of 10 days prior to training and were handled for 1–2 min per day for 5 days preceding the training day.

### Object Recognition Training and Testing Procedures

The experimental apparatus was a gray open-field box (in cm: 40 w × 40 d × 40 h) with the floor covered with sawdust and placed in a dimly illuminated room. The objects to be discriminated were transparent glass vials (5.5 cm diameter and 5 cm height) and white glass light bulbs (6 cm diameter and 11 cm length). The rats were not habituated to the experimental context prior to the training trial. Previously, we have shown that this produces novelty-induced arousal during the training (Okuda et al., 2004). On the training trial, each rat was placed individually in the training apparatus at the opposite end from the objects and was allowed to explore two identical objects (A1 and A2) for 3 min, which by itself is insufficient to induce long-lasting memory of the objects (Okuda et al., 2004; Bermúdez-Rattoni et al., 2005; Roozendaal et al., 2006, 2008). Rats’ behavior was recorded with a video camera positioned above the experimental apparatus. Videos were analyzed off-line by a trained observer blind to treatment condition. The total time spent exploring the two objects during the training trial was taken as a measure of object exploration. Rats showing a total exploration time of <10 s on the training trial were removed from analyses, because previous findings indicated that such rats do not acquire the task (Okuda et al., 2004). To avoid the presence of olfactory cues, the sawdust was stirred and the objects were cleaned with 70% ethanol after each animal.

Some rats were sacrificed for tissue collection 1 h after training and immediate posttraining drug treatment. Other rats were tested for retention 24 h after the training trial. During the retention test, one copy of the familiar object (A3) and a new object (B) were placed in the same location as stimuli during the training trial. All combinations and locations of objects were used in a balanced manner to reduce potential biases due to preference for particular locations or objects. The rat was placed in the experimental apparatus for 3 min and the time spent exploring each object and the total time spent exploring both objects were recorded. Exploration of an object was defined as pointing the nose to the object at a distance of <1 cm and/or touching it with the nose. Turning around, climbing or sitting on an object was not considered as exploration. In order to analyze cognitive performance, a discrimination index was calculated as the difference in time exploring the novel and familiar object, expressed as the ratio of the total time spent exploring both objects (i.e., [Time Novel − Time Familiar/Time Novel + Time Familiar]× 100%).

### Drug Administration

Norepinephrine (1.0 μg; Sigma-Aldrich) was dissolved in saline and administered into the BLA immediately after the object recognition training trial (Roozendaal et al., 2008). Bilateral infusions of drug or an equivalent volume of saline were administered into the BLA via 30-gauge injection needles connected to 10-μl Hamilton microsyringes by polyethylene (PE-20) tubing. The injection needles protruded 2.0 mm beyond the cannula tips and a 0.2-μl injection volume per hemisphere was infused over a period of 30 s by an automated syringe pump (Stoelting Co., Dublin, Ireland). The injection needles were retained within the cannulae for an additional 20 s to maximize diffusion and to prevent backflow of drug into the cannulae. The infusion volume was based on previous findings from our laboratory indicating that drug infusions into the adjacent central amygdala do not affect memory consolidation (Roozendaal and McGaugh, 1996, 1997). Drug solutions were freshly prepared before each experiment.

### Cannula Placement Verification

After object recognition memory testing, rats were deeply anesthetized with sodium pentobarbital (100 mg/kg, ip) and perfused transcardially with 0.9% saline followed by 4% formaldehyde. After decapitation, the brains were removed and immersed in fresh 4% formaldehyde. At least 24 h before sectioning, the brains were transferred to a 30% sucrose solution in saline for cryoprotection. Coronal sections of 50 μm were cut on a cryostat, mounted on gelatin-coated slides, and stained with cresyl violet. The sections were examined under a light microscope and determination of the location of injection needle tips in the BLA was made according to the atlas plates of Paxinos and Watson (2007) by an observer blind to drug treatment condition. Ten rats with injection needle tip placements outside the BLA or with extensive tissue damage at the injection needle site were removed from behavioral analyses.

### Insular Cortex Tissue Collection and Histone Preparation

Rats for the molecular investigations were deeply anesthetized with an overdose of pentobarbital (100 mg/kg, ip) 1 h after training and drug treatment. Within 90 s after the pentobarbital injection, the rats were decapitated, the brains rapidly removed and flash frozen by submersion for 2 min in a beaker filled with pre-cooled isopentane on dry ice. Flash-frozen brains were stored at −80°C until tissue processing. The anterior part of the brain was cut on a cryostat into 350-μm-thick coronal slices for IC tissue collection. The rest of the brain, containing the BLA, was immersed in 4% formaldehyde for at least 3 days, and then transferred to a 30% sucrose solution for cryoprotection. Coronal sections of 50 μm were cut on a cryostat, collected on gelatin-coated slides, and fixed in 100% acetone before staining with cresyl violet. Determination of injection needle placement within the BLA was performed as previously described. Fifteen rats for the molecular experiments were removed from analyses on histological grounds.

IC tissue was dissected from frozen 350-μm-thick coronal slices using a 1.25-mm brain puncher (Stoelting Co., Dublin, Ireland). Bilateral punches from the anterior IC were collected from three consecutive slices to a total of six punches, approximate range of coordinates: AP, +2.7 to −0.3 mm; ML, ±4.0 − 6.0 mm; DV, −5.0 to −7.0 mm. Histones were isolated according to the Levenson et al. (2004) protocol with some modifications. All procedures were performed on ice and all solutions and centrifugations were chilled to 4°C prior to use. Tissue was homogenized in 100 μl of hypotonic lysis buffer [250 mM sucrose, 50 mM Tris, 25 mM KCl, 1 Complete protease inhibitor cocktail tablet (Roche), 1 phosphatase inhibitor tablet (Roche), 0.9 mM NaB, pH 7.5] and grinded for 10 s. The homogenate was centrifuged at 7,800 × g for 1 min. The supernatant (cytoplasmic fraction) was removed and stored at −20°C for determination of pERK1/2 levels. The pellet (nuclear fraction) was resuspended in 100 μl of 0.2 M HCl (Rodriguez-Collazo et al., 2009) for 1 h on ice and vortexed every 10 min, and then centrifuged at 16,000 × g for 15 min. The supernatant was transferred into a fresh tube and 30 μl of trichloroacetic acid containing 4 mg/ml deoxycholic acid was added to precipitate the proteins for 15 min and then centrifuged at 16,000 × g for 15 min. The supernatant was discarded and the pellet was washed with 100 μl of ice-cold acidified acetone (0.1% HCl) for 5 min and then centrifuged at 16,000 × g for 5 min, washed again with 100 μl of ice-cold 100% acetone for 5 min, and centrifuged at 16,000 × g for 5 min. Finally, the supernatant was removed and the remaining histone pellet was dried for 15 min for the remaining acetone to evaporate. The pellet was resuspended in 30 μl of 50 mM Tris (pH 8.0) and sample buffer (5×) was added to prevent over-dilution of the samples. The samples were then boiled and 10-μl aliquots were stored at −80°C to protect histones from degradation (Rumbaugh and Miller, 2011).

### Western Blotting

Histone samples (10 μl) or equal protein concentrations of cytoplasmic samples (5 μg) for pERK1/2 identification were resolved on a discontinuous polyacrylamide gel consisting of a 20% acrylamide resolving gel for histone proteins, or 10% acrylamide resolving gel for ERK1/2 protein, and 4% acrylamide stacking gel. The gel was then blotted onto a polyvinylidene difluoride (PVDF) membrane for immunoblotting (Millipore, Amsterdam, Netherlands). The membranes were blocked for 1 h in blocking buffer (LI-COR Biosciences, Bad Homburg, Germany), diluted 1:1 in phosphate-buffered saline (PBS) [or Tris-buffered saline (TBS) for phospho-antibodies], then incubated with primary antibody overnight at 4°C, followed by incubation with the appropriate secondary antibody for 2 h at room temperature. Primary and secondary antibodies were dissolved in the same blocking buffer. Band intensity was determined and quantified using an Odyssey IR scanner (LI-COR Biosciences). The blot was then stripped and re-probed with antibody against total histone H3 (Levenson et al., 2004) or total mitogen-activated protein kinase (MAPK). Levels of acH3K14, acH2B, acH4, pH3S10 and 3meH3K27 were normalized to total histone H3 levels and pERK1/2 levels were normalized to total MAPK levels for each sample (Patterson et al., 2001; Chwang et al., 2006).

### Antibodies

The primary antibodies and their dilutions are: acetylated histone H3 at lysine 14 (acH3K14; 1:1,000; Millipore), acetylated histone H2B (acH2B; 1:2,000; Millipore), acetylated histone H4 (acH4; 1:1,000; Millipore), phosphorylated histone H3 at serine 10 (pH3S10; 1:1,000; Millipore), tri-methylated histone H3 at lysine 27 (3meH3K27; 1:2,000; Millipore), total H3 (1:2,000; Millipore), phospho-p44/42 MAPK (1:2,000; Cell Signaling Technology, Leiden, Netherlands) and p44/42 MAPK (1:2,000; Cell Signaling Technology). The secondary antibodies were goat anti-rabbit (1:25,000; LI-COR Biosciences) and donkey anti-mouse (1:20,000; LI-COR Biosciences).

### Statistics

Data are expressed as mean ± SEM. The discrimination index and total object exploration time were analyzed with unpaired *t*-tests. One-sample *t*-tests were used to determine whether the discrimination index was different from zero (i.e., chance level) and thus whether memory was expressed. Normalized histone PTM and pERK1/2 data are expressed as the percentage of the mean of the saline-treated home cage control group and analyzed with two-way ANOVAs with training condition and drug treatment as between-subject variables, followed by *post hoc* comparison tests, when appropriate. A probability level of <0.05 was accepted as statistical significance for all tests. The number of rats per group is indicated in the figure legends.

## Results

### Posttraining Norepinephrine Administration into the BLA Enhances the Consolidation of Object Recognition Memory

**Figure 1:**
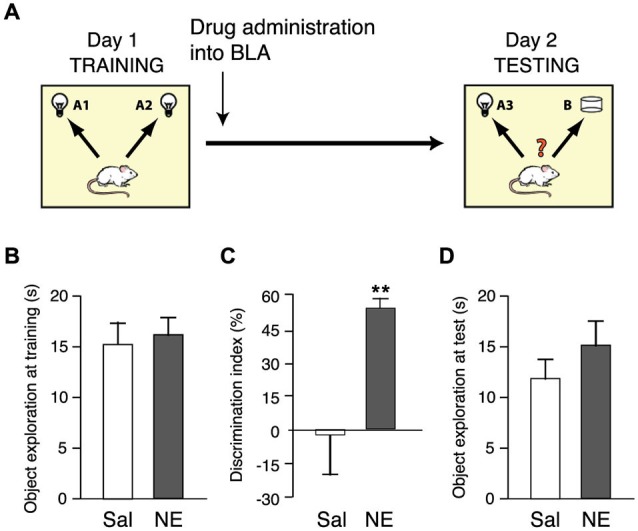
**Norepinephrine administration into the BLA enhances the consolidation of object recognition memory. (A)** Diagram of the experimental procedure. Rats were trained for 3 min on an object recognition task followed immediately by bilateral intra-BLA infusions of norepinephrine (NE; 1.0 μg in 0.2 μl; *n* = 9) or saline (Sal; *n* = 8). **(B)** Total exploration time during the 3-min training trial, before drug treatment, did not differ between groups. **(C)** Norepinephrine significantly increased the discrimination index on the 24-h retention test. **(D)** Total exploration time during the retention test did not differ between groups. Data are shown as mean ± SEM. **p < 0.01.

We first examined the effect of norepinephrine administration into the BLA after object recognition training on memory consolidation. For this, rats were trained on the object recognition task for 3 min and immediately after the training trial given bilateral infusions of norepinephrine (1.0 μg in 0.2 μl) or saline into the BLA. To determine whether animals exhibit a long-term memory for the object seen during the training trial, rats were given a 24-h retention test in which one object was familiar and the other object was novel. If the animal generates a long-term memory for the familiar object, it will spend significantly more time exploring the novel object during the retention test. Figure 1A shows a schematic diagram of the experimental design.

Norepinephrine treatment immediately after the training trial enhanced long-term memory for the familiar object. As shown in Figure 1B, the total time spent exploring the two identical objects during the 3-min training trial, before drug treatment, did not differ between groups (*p* = 0.65). Figure 1C shows the discrimination index during the 24 h retention test trial. One-sample *t*-test revealed that the discrimination index of saline-treated control rats did not differ significantly from zero (i.e., chance level, *t*_7_ = −0.16; *p* = 0.88), indicating that they did not show any evidence of retention of the training. Norepinephrine administration into the BLA immediately after object recognition training significantly enhanced the discrimination index (*p* < 0.01). Moreover, one-sample *t*-test indicated that norepinephine-treated rats exhibited a significant exploration preference for the novel object (*t*_8_ = 10.78; *p* < 0.0001). The total time spent exploring the two objects during the retention test trial did not differ between groups (*p* = 0.31; Figure 1D), indicating that the drug infusion did not induce a general change in the rats’ incentive to explore the objects.

**Figure 2:**
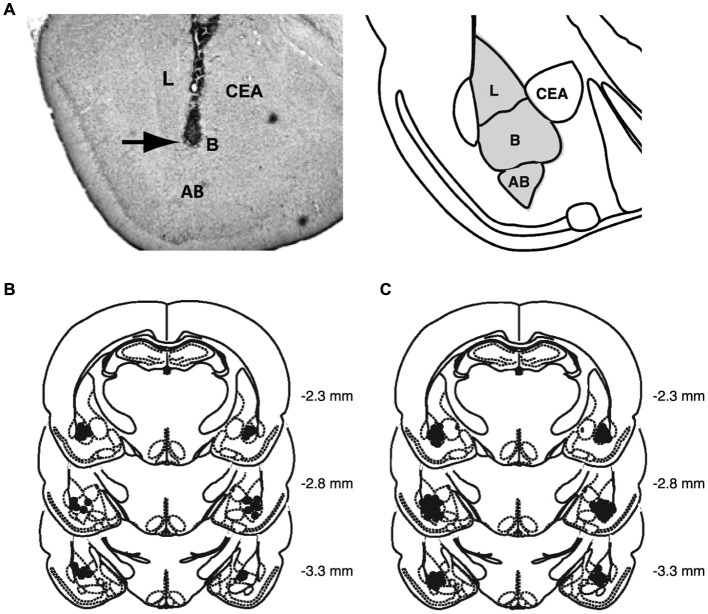
**Histological analyses. (A)** Representative photomicrograph illustrating placement of a cannula and needle tip terminating in the BLA and a diagram representing the different nuclei of the BLA, the lateral nucleus (L), basal nucleus (B), and accessory basal nucleus (AB), and central nucleus (CEA). Arrow points to needle tip. **(B)** The location of needle tips within the BLA of all rats included in the analysis for the behavioral study. **(C)** The location of needle tips within the BLA of all rats included in the analysis for the molecular study.

Figure 2 shows a representative photomicrograph of an infusion needle tip terminating within the BLA as well as the histological analyses of the infusion needle tip placements of all rats included in the analysis.

### A Memory-Enhancing Dose of Norepinephrine Administered into the BLA After Object Recognition Training Reduces Histone Acetylation Levels in the IC

To determine whether this memory-enhancing dose of norepinephrine administered into the BLA after object recognition training triggers changes in the chromatin state in the IC, we examined changes in the following histone markers: acetylation of histone H3 at lysine 14 (acH3K14), acetylation of histone H2B (acH2B) and acetylation of histone H4 (acH4), as well as phosphorylation of histone H3 at serine 10 (pH3S10) and tri-methylation of histone H3 at lysine 27 (3meH3K27). Some rats received norepinephrine (1.0 μg in 0.2 μl) or saline into the BLA immediately after 3 min of object recognition training. Other groups of rats received the same drug infusions without training. For both groups, changes in histone markers in the IC were assessed 1 h after drug treatment. Changes in histone PTMs, normalized to total histone H3 levels, are shown as percentage (mean ± SEM) relative to saline-treated home cage control rats. We also investigated whether the norepinephrine administration altered pERK1/2 levels in the IC 1 h after training. Changes in pERK1/2 levels, normalized to total MAPK levels, are also shown as percentage of the saline-treated home cage control rats. Figure 2C shows histological analysis of injection needle tip placement in the BLA of all rats included in the analysis for the molecular experiments. Total exploration time of the two identical objects during the 3-min training trial, before drug treatment, did not differ between groups (*p* = 0.19).

**Figure 3:**
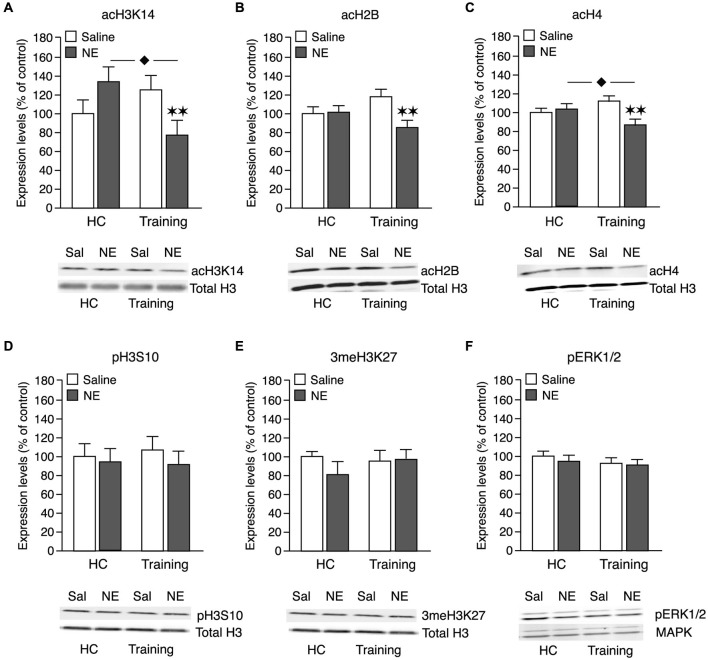
**Effect of a memory-enhancing dose of norepinephrine administered into the BLA on histone PTMs and pERK1/2 activity in the IC**. Rats were trained on an object recognition task for 3 min and given posttraining bilateral infusions of a memory-enhancing dose of norepinephrine (NE; 1.0 μg in 0.2 μl) or saline (Sal) control into the BLA. Other groups of rats received the same dose of norepinephrine or saline into the BLA without training (home cage; HC). Tissue was collected 1 h after training and drug administration and histones were prepared from punches taken from the IC. Histone PTMs were revealed by immunoblotting and normalized to total histone H3 levels on the same blot. Histone PTMs (mean ± SEM) are shown as percentage of the saline-treated home cage control group. Quantification of acH3K14 (**A**, Sal-HC: *n* = 13; NE-HC: *n* = 10; Sal-Training: *n* = 10; NE-Training: *n* = 11), acH2B (**B**, Sal-HC: *n* = 13; NE-HC: *n* = 10; Sal-Training: *n* = 10; NE-Training: *n* = 11), acH4 (**C**, Sal-HC: *n* = 13; NE-HC: *n* = 11; Sal-Training: *n* = 10; NE-Training: *n* = 11), pH3S10 (**D**, Sal-HC: *n* = 13; NE-HC: *n* = 11; Sal-Training: *n* = 10; NE-Training: *n* = 12) and 3meH3K27 (**E**, Sal-HC: *n* = 12; NE-HC: *n* = 11; Sal-Training: *n* = 11; NE-Training: *n* = 11) levels in home cage controls and trained rats. Levels of total H3 remained unchanged in the different experimental conditions. **(F)** Cytoplasmic fractions of the IC were prepared for pERK1/2 assessment. pERK1/2 was revealed by immunoblotting and normalized to total MAPK levels. Sal-HC: *n* = 13; NE-HC: *n* = 9; Sal-Training: *n* = 11; NE-Training: *n* = 12. **p < 0.01 vs. the trained saline group. ^⧫^ *p* < 0.05 vs. the non-trained norepinephrine group.

Our findings indicate that this memory-enhancing dose of norepinephrine administered into the BLA after the training experience induced a global reduction in histone acetylation, whereas it did not alter the phosphorylation or methylation state of the histone molecules. pERK1/2 levels also remained unchanged. Norepinephrine administered into the BLA of home cage control animals did not induce any changes in histone PTMs or pERK1/2 levels. As shown in Figure 3A, two-way ANOVA for acH3K14 levels indicated no main norepinephrine (*F*_1,40_ = 1.10; N.S.) or training effect (*F*_1,40_ = 0.19; N.S.), but a significant interaction between both factors (*F*_1,40_ = 7.38; *p* < 0.01). Norepinephrine infused into the BLA of home cage control rats did not change acH3K14 levels in the IC 1 h later when compared to non-trained saline control rats (*p* = 0.67), indicating that the norepinephrine administration alone is insufficient to alter acH3K14 levels within the IC. The 3-min object recognition training session by itself, which is not sufficient to induce long-term memory, also did not significantly change acH3K14 levels when compared to home cage control rats (*p* = 0.14). However, norepinephrine infusions into the BLA after object recognition training significantly reduced acH3K14 levels in the IC when compared to saline-treated trained rats (*p* < 0.01) as well as when compared to norepinephrine-treated home cage control rats (*p* < 0.05). Thus, these findings indicate that norepinephrine selectively decreased acH3K14 levels in the IC in the context of object recognition training.

As shown in Figure 3B, two-way ANOVA for acH2B levels indicated a significant norepinephrine effect (*F*_1,40_ = 4,70; *p* < 0.05), no main training effect (*F*_1,40_ = 0.01; N.S.), but a significant interaction between both factors (*F*_1,40_ = 5,22; *p* < 0.05). Highly comparable to the effect on acH3K14, norepinephrine infused into the BLA of home cage control rats did not change acH2B levels within the IC (*p* = 0.93). The object recognition training alone also did not significantly alter acH2B levels (*p* = 0.12). However, posttraining administration of norepinephrine into the BLA after object recognition training significantly reduced acH2B levels in the IC when compared to saline-treated trained rats (*p* < 0.01).

As shown in Figure 3C, two-way ANOVA for acH4 levels indicated an almost significant norepinephrine effect (*F*_1,41_ = 3.72; *p* = 0.06) whereas the object recognition training did not have any effect (*F*_1,41_ = 0.08; N.S.). Most importantly, there was a significant interaction effect between norepinephrine treatment and training (*F*_1,41_ = 7.53; *p* < 0.01). Intra-BLA norepinephrine administration to non-trained control rats did not change acH4 levels in the IC (*p* = 0.53). In contrast, norepinephrine infused into the BLA after object recognition training significantly reduced acH4 levels when compared to saline-treated trained rats (*p* < 0.01) or norepinephrine-treated home cage control rats (*p* < 0.05).

Figure 3D shows pH3S10 levels. Two-way ANOVA revealed no norepinephrine (*F*_1,42_ = 0.47; N.S.), training (*F*_1,42_ = 0.02; N.S.) or interaction effect (*F*_1,42_ = 0.10; N.S.). Thus, these findings indicate that the norepinephrine administration did not change pH3S10 levels in either trained or non-trained animals.

Figure 3E shows that 3meH3K27 levels also remained unchanged. Two-way ANOVA revealed no significant norepinephrine (*F*_1,41_ = 0.53; N.S.), training (*F*_1,41_ = 0.30; N.S.) or interaction effect (*F*_1,41_ = 0.67; N.S.). Thus, these findings indicate that the norepinephrine administration did not change 3meH3K27 levels in either trained or non-trained animals.

As shown in Figure 3F, two-way ANOVA for pERK1/2 levels revealed no norepinephrine (*F*_1,41_ = 1.42; N.S.) or training effect (*F*_1,41_ = 0.05; N.S.), or interaction between the two factors (*F*_1,41_ = 0.40; N.S.). Thus, these findings indicate that the norepinephrine administration also did not change pERK1/2 levels in either trained or non-trained animals.

## Discussion

This study was aimed at investigating whether a memory-enhancing dose of norepinephrine administered into the BLA after object recognition training induces chromatin modification in the IC. In this experiment we trained rats on an object recognition task and created an “arousal-like” situation by administering norepinephrine into the BLA after the training trial. Consistent with earlier findings (Roozendaal et al., 2008), this norepinephrine infusion enhanced the consolidation of object recognition memory. To determine whether the “arousal-like situation” triggers chromatin alterations in the IC, we assessed a battery of histone PTMs known to be involved in neural plasticity and memory formation (Levenson et al., 2004; Chwang et al., 2006; Fischer et al., 2007; Koshibu et al., 2009, 2011; Bousiges et al., 2010; Gräff et al., 2012). We observed that this memory-enhancing dose of norepinephrine reduced the acetylation levels of H3K14 as well as of H2B and H4 in the IC 1 h after object recognition training. These effects were specific to trained rats, as norepinephrine infusions into the BLA of home cage control animals did not induce any changes in these histone markers. pH3S10 and 3meH3K27 levels were not altered by either the norepinephrine infusion or object recognition training alone.

It has long been known that noradrenergic activation of the BLA mediates emotional arousal effects on the consolidation of long-term memory (McGaugh, 2000, 2004; McGaugh and Roozendaal, 2002). Extensive evidence indicates that such noradrenergic activation of the BLA enhances memory consolidation of different training experiences by facilitating time-dependent information storage processes in other brain regions, including the hippocampus, caudate nucleus and cortical areas (Packard et al., 1994; McGaugh, 2004; McIntyre et al., 2005). However, the molecular mechanism(s) underlying this BLA influence on information storage processes in its target regions remain to be elucidated. Our current finding that norepinephrine administration into the BLA after object recognition training induced a global reduction in histone acetylation, without altering the phosphorylation and methylation state, provides novel evidence indicating that arousal-associated BLA activity induces training-specific changes in histone PTMs in the IC (and likely other target areas). However, the pattern of histone PTM changes that we observed in the current study was rather unexpected. To date, most studies have linked a histone hyperacetylated state and decondensed chromatin structure with facilitated transcription, resulting in enhanced synaptic plasticity and long-term memory (Levenson et al., 2004; Chwang et al., 2006; Barrett and Wood, 2008; Gupta et al., 2010; Roozendaal et al., 2010). Previously, we found that inducing a hyperacetylated state within the IC by local posttraining administration of the HDAC inhibitor NaB enhanced object recognition memory (Roozendaal et al., 2010). Findings in the literature show that the nature and extent of specific histone PTM marks can vary considerably, depending on the memory task, brain region as well as other experimental parameters. For example, hyperacetylation of H3K14, but not H4, was observed in the CA1 subregion of the hippocampus 1 h after contextual fear conditioning (Levenson et al., 2004). On the other hand, spatial training in a water maze increased acetylation of H2B and H4 in the hippocampus, but did not affect the acetylation of H3K14 (Bousiges et al., 2010). In another study, the hyperacetylation of H3K14 after contextual fear conditioning was associated with an increased phosphorylation of H3S10 (Chwang et al., 2006). Gräff et al. (2012) have also shown the combined acetylation of H3K14 and phosphorylation of H3S10 in the hippocampus after object recognition training. In the present study, we observed that the norepinephrine administration into the BLA did not produce the expected hyperacetylation. In fact, acetylation levels of H3K14 as well as that of H2B and H4 were significantly reduced 1 h after the training experience and drug administration, whereas the phosphorylation of histone H3S10 and tri-methylation of H3K27 levels were not altered. The only case where such a decrease in histone acetylation was previously demonstrated was in chronic stress (Ferland and Schrader, 2011). One study investigating histone acetylation changes after chronic social defeat indicated a transient decrease, followed by a persistent increase, in acH3K14 levels in the nucleus accumbens (Covington et al., 2009). The persistent increase in acetylation was associated with a reduction in histone deacetylase 2 enzyme activity (Covington et al., 2011). Our findings further indicate that the norepinephrine infusion did not alter pERK1/2 activity in the IC 1 h later. ERK1/2 activation has been demonstrated to be critical for histone H3 acetylation and phosphorylation in contextual fear conditioning in the CA1 region of hippocampus (Levenson et al., 2004; Chwang et al., 2006) as well as in stress conditions such as forced swimming that induces phospho-acetylation of H3 (Chandramohan et al., 2008; Gutièrrez-Mecinas et al., 2011; Mifsud et al., 2011). Although we cannot exclude that this 1-h time point may have not been optimal to capture the peak of the pERK1/2 response (Kobayashi et al., 2010), previous studies reported that phosphorylation of ERK1/2 was found in the IC 2–6 h after novel taste learning (Swank and Sweatt, 2001) or 1 h after contextual fear conditioning in the CA1 area of the hippocampus (Levenson et al., 2004).

Although it is currently unknown how a more extensive object recognition training experience, resulting in good 24-h memory, would affect histone PTMs and gene transcription in the IC, our observation that a memory-enhancing dose of norepinephrine reduced histone acetylation levels, and possibly consequent changes in transcriptional activity, within the IC is rather puzzling and does not seem to concur with prior evidence that direct administration of a protein-synthesis inhibitor into the IC impairs long-term memory of object recognition training (Balderas et al., 2008). Moreover, in a previous study we found that systemic administration of the stress hormone corticosterone increased acH3K14 levels in the IC 1 h following training on an object recognition task and enhanced the consolidation of object recognition memory. These findings indicate that although both systemic corticosterone and intra-BLA administration of norepinephrine have the same behavioral outcome, i.e., an enhancement of object recognition memory, they were associated with opposite effects on histone acetylation within the IC. It is plausible that these differential molecular effects may be due to the different routes of drug administration: The systemically administered corticosterone could act directly in the IC to induce chromatin remodeling, whereas intra-BLA administration of norepinephrine must induce chromatin remodeling in the IC indirectly via neural pathways and network changes. It is not unlikely that norepinephrine administration into the BLA induces rapid changes in network properties, which might be associated with fast changes in histone acetylation. As histone acetylation-deacetylation is a highly dynamic process [e.g., NaB administration to a mammalian non-neuronal cell culture induced a fast hyperacetylation of core histones (*t*_1/2_ = 3–7 min) (Davie, 2003)], it is possible that the norepinephrine administration after object recognition training first induced a rapid increase in histone acetylation within the IC followed by deacetylation at 1 h. On the other hand, it should be noted that the exact role of the IC as part of the broader emotional learning and memory network is largely unknown and might be associated with decreased neural activity. For example, we showed in a previous study that systemic administration of a memory-enhancing dose of corticosterone after inhibitory avoidance training resulted in a rapid decrease in the number of pERK1/2-positive pyramidal neurons within the IC (Fornari et al., 2012b). Furthermore, a recent functional magnetic resonance imaging study in humans showed that the combined oral administration of cortisol and the noradrenergic stimulant yohimbine shortly before the encoding of emotionally arousing pictures shifted brain activation patterns and led to a strong deactivation of the IC, along with the hippocampus and orbitofrontal cortex (van Stegeren et al., 2010). Moreover, the magnitude of this deactivation correlated with enhanced recall of the material when assessed 1 week later. It is possible that a reduced overall activity of the IC (and other frontal areas) during these conditions could reflect either a loss of top-down inhibition, and therefore activation (disinhibition) of other brain regions, or an increased signal-to-noise ratio, resulting in an increased detection of novel or relevant stimuli and enhancing the consolidation of memory of arousing experiences (Menon and Uddin, 2010; van Stegeren et al., 2010).

Another intriguing possibility is that the primary role of BLA noradrenergic activation is to activate transcription factors and coactivators, which then could interact with chromatin modification mechanisms in its target regions. It is well established that the consequence of changes in histone PTMs on transcriptional activity depends on an intimate interaction with a large number of transcription factors and coactivators (Vecsey et al., 2007). As indicated, in a previous study we demonstrated that direct administration of the HDAC inhibitor NaB into the IC enhanced the consolidation of object recognition memory (Roozendaal et al., 2010). However, co-administration of a glucocorticoid receptor (GR) antagonist or cAMP-dependent protein kinase (PKA) inhibitor completely abolished the effect of the HDAC inhibitor on memory enhancement. These findings indicate that inducing a histone hyperacetylated state via HDAC inhibition is not sufficient to enhance long-term memory. It is still necessary to have upstream arousal-associated signaling via GR and PKA activity. Presumably, these signaling events are triggering steps necessary to activate transcription factors and coactivators such as cAMP response-element binding (CREB) protein and CREB-binding protein (CBP; Roozendaal et al., 2010). Recently, we found that the β-adrenoceptor antagonist propranolol administered into the BLA after object recognition training did not prevent the effect of systemic NaB administration on hyperacetylation of H3K14 in the IC (Beldjoud et al., unpublished observation). However, the propranolol completely abolished the NaB-induced memory enhancement. These findings are similar to those of another study (Blank et al., 2014) indicating that temporary inactivation of the BLA with muscimol blocks the enhancement of inhibitory avoidance memory induced by HDAC inhibitor infusions into the hippocampus. These findings suggest that BLA (noradrenergic) activity might not directly alter histone acetylation mechanisms, but that it provides an additional obligatory factor, such as the activation of transcription factors and coactivators, that interacts with the chromatin remodeling changes in regulating gene transcription and neural plasticity. The currently observed reduction in acetylation levels should, in this case, not necessarily be interpreted as a direct effect of the BLA stimulation but might instead be an indirect consequence of feedback regulation mechanisms due to elevated transcription factor levels. In this perspective, mapping the genome-wide location of specific histone marks and transcription factors using chromatin immunoprecipitation (ChIP) will offer a more detailed understanding of the effect of noradrenergic activation of the BLA on chromatin modification mechanisms in influencing gene expression and may significantly contribute to our understanding of why emotionally arousing experiences are well remembered.

## Conflict of Interest Statement

The authors declare that the research was conducted in the absence of any commercial or financial relationships that could be construed as a potential conflict of interest.

## References

[B1] Albasser M. M. Davies M. Futter J. E. Aggleton J. P. (2009). Magnitude of the object recognition deficit associated with perirhinal cortex damage in rats: effects of varying the lesion extent and the duration of the sample period. Behav. Neurosci. 123, 115–124. 10.1037/a0013829 19170436

[B2] Balderas I. Rodriguez-Ortiz C. J. Salgado-Tonda P. Chavez-Hurtado J. McGaugh J. L. Bermúdez-Rattoni F. (2008). The consolidation of object and context recognition memory involve different regions of the temporal lobe. Learn. Mem. 15, 618–624. 10.1101/lm.1028008 18723431 PMC2632790

[B3] Barker G. R. Warburton E. C. (2011). When is the hippocampus involved in recognition memory? J. Neurosci. 31, 10721–10731. 10.1523/JNEUROSCI.6413-10.2011 21775615 PMC6622630

[B4] Barrett R. M. Wood M. A. (2008). Beyond transcription factors: the role of chromatin modifying enzymes in regulating transcription required for memory. Learn. Mem. 15, 460–467. 10.1101/lm.917508 18583646 PMC3960025

[B5] Barsegyan A. McGaugh J. L. Roozendaal B. (2014). Noradrenergic activation of the basolateral amygdala modulates the consolidation of object-in-context recognition memory. Front. Behav. Neurosci. 8:160. 10.3389/fnbeh.2014.00160 24847228 PMC4021114

[B7] Berman D. E. Hazvi S. Neduva V. Dudai Y. (2000). The role of identified neurotransmitter systems in the response of insular cortex to unfamiliar taste: activation of ERK1–2 and formation of a memory trace. J. Neurosci. 20, 7017–7023. 10995847 10.1523/JNEUROSCI.20-18-07017.2000 PMC6772803

[B8] Bermúdez-Rattoni F. (2004). Molecular mechanisms of taste-recognition memory. Nat. Rev. Neurosci. 5, 209–217. 10.1038/nrn1344 14976520

[B9] Bermúdez-Rattoni F. (2014). The forgotten insular cortex: its role on recognition memory formation. Neurobiol. Learn. Mem. 109, 207–216. 10.1016/j.nlm.2014.01.001 24406466

[B10] Bermúdez-Rattoni F. Introini-Collison I. B. McGaugh J. L. (1991). Reversible inactivation of the insular cortex by tetrodotoxin produces retrograde and anterograde amnesia for inhibitory avoidance and spatial learning. Proc. Natl. Acad. Sci. U S A 88, 5379–5382. 10.1073/pnas.88.12.5379 2052615 PMC51876

[B11] Bermúdez-Rattoni F. McGaugh J. L. (1991). Insular cortex and amygdala lesions differentially affect acquisition on inhibitory avoidance and conditioned taste aversion. Brain Res. 549, 165–170. 10.1016/0006-8993(91)90616-4 1654172

[B12] Bermúdez-Rattoni F. Okuda S. Roozendaal B. McGaugh J. L. (2005). Insular cortex is involved in consolidation of object recognition memory. Learn. Mem. 12, 447–449. 10.1101/lm.97605 16166398

[B13] Blank M. Dornelles A. S. Werenicz A. Velho L. A. Pinto D. F. Fedi A. C.. (2014). Basolateral amygdala activity is required for enhancement of memory consolidation produced by histone deacetylase inhibition in the hippocampus. Neurobiol. Learn. Mem. 111, 1–8. 10.1016/j.nlm.2014.02.009 24583371

[B14] Bousiges O. de Vasconcelos A. P. Neidl R. Cosquer B. Herbeaux K. Panteleeva I.. (2010). Spatial memory consolidation is associated with induction of several lysine-acetyltransferase (histone acetyltransferase) expression levels and H2B/H4 acetylation-dependent transcriptional events in the rat hippocampus. Neuropsychopharmacology 35, 2521–2537. 10.1038/npp.2010.117 20811339 PMC3055563

[B15] Chandramohan Y. Droste S. K. Arthur J. S. Reul J. M. (2008). The forced swimming-induced behavioural immobility response involves histone H3 phospho-acetylation and c-Fos induction in dentate gyrus granule neurons via activation of the N-methyl-d-aspartate/extracellular kinase signalling pathway. Eur. J. Neurosci. 27, 2701–2713. 10.1111/j.1460-9568.2008.06230.x 18513320

[B16] Chwang W. B. O’Riordan K. J. Levenson J. M. Sweatt J. D. (2006). ERK/MAPK regulates hippocampal histone phosphorylation following contextual fear conditioning. Learn. Mem. 13, 322–328. 10.1101/lm.152906 16741283 PMC1475813

[B17] Covington H. E. 3rd Maze I. LaPlant Q. C. Vialou V. F. Ohnishi Y. N. Berton O.. (2009). Antidepressant actions of histone deacetylase inhibitors. J. Neurosci. 29, 11451–11460. 10.1523/JNEUROSCI.1758-09.2009 19759294 PMC2775805

[B18] Covington H. E. 3rd Vialou V. F. Laplant Q. Ohnishi Y. N. Nestler E. J. (2011). Hippocampal-dependent antidepressant-like activity of histone deacetylase inhibition. Neurosci. Lett. 493, 122–126. 10.1016/j.neulet.2011.02.022 21335060 PMC3074929

[B19] Davie J. R. (2003). Inhibition of histone deacetylase activity by butyrate. J. Nutr. 113, 2485S–2493S. 12840228 10.1093/jn/133.7.2485S

[B20] Ennaceur A. Delacour J. (1988). A new one-trial test for neurobiological studies of memory in rats. 1: behavioral data. Behav. Brain Res. 31, 47–59. 10.1016/0166-4328(88)90157-x 3228475

[B21] Escobar M. L. Chao V. Bermúdez-Rattoni F. (1998). In vivo long-term potentiation in the insular cortex: NMDA receptor dependence. Brain Res. 779, 314–319. 10.1016/s0006-8993(97)01175-x 9473708

[B22] Ferland C. L. Schrader L. A. (2011). Regulation of histone acetylation in the hippocampus of chronically stressed rats: a potential role of sirtuins. Neuroscience 174, 104–114. 10.1016/j.neuroscience.2010.10.077 21056634 PMC3020273

[B23] Ferry B. Roozendaal B. McGaugh J. L. (1999). Basolateral amygdala noradrenergic influences on memory storage are mediated by an interaction between beta- and alpha1-adrenoceptors. J. Neurosci. 19, 5119–5123. 10366644 10.1523/JNEUROSCI.19-12-05119.1999 PMC6782651

[B24] Fischer A. Sananbenesi F. Wang X. Dobbin M. Tsai L. H. (2007). Recovery of learning and memory is associated with chromatin remodelling. Nature 447, 178–182. 10.1038/nature05772 17468743

[B25] Fornari R. V. Wichmann R. Atsak P. Atucha E. Barsegyan A. Beldjoud H.. (2012a). Rodent stereotaxic surgery and animal welfare outcome improvements for behavioral neuroscience. J. Vis. Exp. 59:e3528. 10.3791/3528 22314779 PMC3353515

[B26] Fornari R. V. Wichmann R. Atucha E. Desprez T. Eggens-Meijer E. Roozendaal B. (2012b). Involvement of the insular cortex in regulating glucocorticoid effects on memory consolidation of inhibitory avoidance training. Front. Behav. Neurosci. 6:10. 10.3389/fnbeh.2012.00010 22435055 PMC3304473

[B27] Gräff J. Woldemichael B. T. Berchtold D. Dewarrat G. Mansuy I. M. (2012). Dynamic histone marks in the hippocampus and cortex facilitate memory consolidation. Nat. Commun. 3:991. 10.1038/ncomms1997 22871810

[B29] Griggs E. M. Young E. J. Rumbaugh G. Miller C. A. (2013). MicroRNA-182 regulates amygdala-dependent memory formation. J. Neurosci. 33, 1734–1740. 10.1523/JNEUROSCI.2873-12.2013 23345246 PMC3711533

[B30] Gupta S. Kim S. Y. Artis S. Molfese D. L. Schumacher A. Sweatt J. D.. (2010). Histone methylation regulates memory formation. J. Neurosci. 30, 3589–3599. 10.1523/JNEUROSCI.3732-09.2010 20219993 PMC2859898

[B31] Gutièrrez-Mecinas M. Trollope A. F. Collins A. Morfett H. Hesketh S. A. Kersanté F.. (2011). Long-lasting behavioral responses to stress involve a direct interaction of glucocorticoid receptors with ERK1/2-MSK1-Elk-1 signaling. Proc. Natl. Acad. Sci. U S A 108, 13806–13811. 10.1073/pnas.1104383108 21808001 PMC3158237

[B32] Haettig J. Stefanko D. P. Multani M. L. Figueroa D. X. McQuown S. C. Wood M. A. (2011). HDAC inhibition modulates hippocampus-dependent long-term memory for object location in a CBP-dependent manner. Learn. Mem. 18, 71–79. 10.1101/lm.1986911 21224411 PMC3032579

[B33] Hatfield T. McGaugh J. L. (1999). Norepinephrine infused into the basolateral amygdala posttraining enhances retention in a spatial water maze task. Neurobiol. Learn. Mem. 71, 232–239. 10.1006/nlme.1998.3875 10082642

[B34] Huff N. C. Wright-Hardesty K. J. Higgins E. A. Matus-Amat P. Rudy J. W. (2005). Context pre-exposure obscures amygdala modulation of contextual-fear conditioning. Learn. Mem. 12, 456–460. 10.1101/lm.6705 16204200

[B35] Hunter R. G. McCarthy K. J. Milne T. A. Pfaff D. W. McEwen B. S. (2009). Regulation of hippocampal H3 histone methylation by acute and chronic stress. Proc. Natl. Acad. Sci. U S A 106, 20912–20917. 10.1073/pnas.0911143106 19934035 PMC2791599

[B36] Introini-Collison I. B. Miyazaki B. McGaugh J. L. (1991). Involvement of the amygdala in the memory-enhancing effects of clenbuterol. Psychopharmacology (Berl) 104, 541–544. 10.1007/bf02245663 1780426

[B37] Kobayashi M. Fujita S. Takei H. Song L. Chen S. Suzuki I.. (2010). Functional mapping of gustatory neurons in the insular cortex revealed by pERK-immunohistochemistry and in vivo optical imaging. Synapse 64, 323–334. 10.1002/syn.20731 19957366

[B38] Koshibu K. Gräff J. Beullens M. Heitz F. D. Berchtold D. Russig H.. (2009). Protein phosphatase 1 regulates the histone code for long-term memory. J. Neurosci. 29, 13079–13089. 10.1523/JNEUROSCI.3610-09.2009 19828821 PMC6665317

[B39] Koshibu K. Gräff J. Mansuy I. M. (2011). Nuclear protein phosphatase-1: an epigenetic regulator of fear memory and amygdala long-term potentiation. Neuroscience 173, 30–36. 10.1016/j.neuroscience.2010.11.023 21093547

[B40] Kye M. J. Neveu P. Lee Y.-S. Zhou M. Steen J. A. Sahin M.. (2011). NMDA mediated contextual conditioning changes miRNA expression. PLoS One 6:e24682. 10.1371/journal.pone.0024682 21931811 PMC3171446

[B41] LaLumiere R. T. Buen T. V. McGaugh J. L. (2003). Post-training intra-basolateral amygdala infusions of norepinephrine enhance consolidation of memory for contextual fear conditioning. J. Neurosci. 23, 6754–6758. 12890768 10.1523/JNEUROSCI.23-17-06754.2003 PMC6740722

[B42] Levenson J. M. O’Riordan K. J. Brown K. D. Trinh M. A. Molfese D. L. Sweatt J. D. (2004). Regulation of histone acetylation during memory formation in the hippocampus. J. Biol. Chem. 279, 40545–40559. 10.1074/jbc.m402229200 15273246

[B44] McDonald A. J. Jackson T. R. (1987). Amygdaloid connections with posterior insular and temporal cortical areas in the rat. J. Comp. Neurol. 262, 59–77. 10.1002/cne.902620106 2442208

[B45] McGaugh J. L. (2000). Memory–a century of consolidation. Science 287, 248–251. 10.1126/science.287.5451.248 10634773

[B46] McGaugh J. L. (2004). The amygdala modulates the consolidation of memories of emotionally arousing experiences. Annu. Rev. Neurosci. 27, 1–28. 10.1146/annurev.neuro.27.070203.144157 15217324

[B47] McGaugh J. L. Roozendaal B. (2002). Role of adrenal stress hormones in forming lasting memories in the brain. Curr. Opin. Neurobiol. 12, 205–210. 10.1016/s0959-4388(02)00306-9 12015238

[B48] McIntyre C. K. Miyashita T. Setlow B. Marjon K. D. Steward O. Guzowski J. F.. (2005). Memory-influencing intra-basolateral amygdala drug infusions modulate expression of Arc protein in the hippocampus. Proc. Natl. Acad. Sci. U S A 102, 10718–10723. 10.1073/pnas.0504436102 16020527 PMC1175582

[B49] Menon V. Uddin L. Q. (2010). Saliency, switching, attention and control: a network model of insula function. Brain Struct. Funct. 214, 655–667. 10.1007/s00429-010-0262-0 20512370 PMC2899886

[B50] Mifsud K. R. Gutièrrez-Mecinas M. Trollope A. F. Collins A. Saunderson E. A. Reul J. M. (2011). Epigenetic mechanisms in stress and adaptation. Brain Behav. Immun. 25, 1305–1315. 10.1016/j.bbi.2011.06.005 21704151

[B51] Miller C. A. Campbell S. L. Sweatt J. D. (2008). DNA methylation and histone acetylation work in concert to regulate memory formation and synaptic plasticity. Neurobiol. Learn. Mem. 89, 599–603. 10.1016/j.nlm.2007.07.016 17881251 PMC2430891

[B52] Miller C. A. Gavin C. F. White J. A. Parrish R. R. Honasoge A. Yancey C. R.. (2010). Cortical DNA methylation maintains remote memory. Nat. Neurosci. 13, 664–666. 10.1038/nn.2560 20495557 PMC3043549

[B54] Miranda M. I. McGaugh J. L. (2004). Enhancement of inhibitory avoidance and conditioned taste aversion memory with insular cortex infusions of 8-Br-cAMP: involvement of the basolateral amygdala. Learn. Mem. 11, 312–317. 10.1101/lm.72804 15169861 PMC419734

[B55] Moraga-Amaro R. Stehberg J. (2012). “The insular cortex and the amygdala: shared functions and interactions,” in The Amygdala–A Discrete Multitasking Manager, ed Ferry B. (Rijeka: InTech), 12–19.

[B56] Nerad L. Ramírez-Amaya V. Ormsby C. E. Bermúdez-Rattoni F. (1996). Differential effects of anterior and posterior insular cortex lesions on the acquisition of conditioned taste aversion and spatial learning. Neurobiol. Learn. Mem. 66, 44–50. 10.1006/nlme.1996.0042 8661250

[B57] Núñez-Jaramillo L. Ramírez-Lugo L. Herrera-Morales W. Miranda M. I. (2010). Taste memory formation: latest advances and challenges. Behav. Brain Res. 207, 232–248. 10.1016/j.bbr.2009.10.040 19891988

[B58] Okuda S. Roozendaal B. McGaugh J. L. (2004). Glucocorticoid effects on object recognition memory require training-associated emotional arousal. Proc. Natl. Acad. Sci. U S A 101, 853–858. 10.1073/pnas.0307803100 14711996 PMC321770

[B59] Packard M. G. Cahill L. McGaugh J. L. (1994). Amygdala modulation of hippocampal-dependent and caudate nucleus-dependent memory processes. Proc. Natl. Acad. Sci. U S A 91, 8477–8481. 10.1073/pnas.91.18.8477 8078906 PMC44629

[B60] Paré D. Smith Y. Paré J. F. (1995). Intra-amygdaloid projections of the basolateral and basomedial nuclei in the cat: Phaseolus vulgaris-leucoagglutinin anterograde tracing at the light and electron microscopic level. Neuroscience 69, 567–583. 10.1016/0306-4522(95)00272-k 8552250

[B61] Patterson S. L. Pittenger C. Morozov A. Martin K. C. Scanlin H. Drake C.. (2001). Some forms of cAMP-mediated long-lasting potentiation are associated with release of BDNF and nuclear translocation of phospho-MAP kinase. Neuron 32, 123–140. 10.1016/S0896-6273(01)00443-3 11604144

[B62] Paxinos G. Watson C. (2007). The Rat Brain in Stereotaxic Coordinates. 6th Edn. San Diego, CA: Academic Press.

[B63] Reolon G. K. Maurmann N. Werenicz A. Garcia V. A. Schröder N. Wood M. A.. (2011). Posttraining systemic administration of the histone deacetylase inhibitor sodium butyrate ameliorates aging-related memory decline in rats. Behav. Brain Res. 221, 329–332. 10.1016/j.bbr.2011.03.033 21421011 PMC3093300

[B64] Rodriguez-Collazo P. Leuba S. H. Zlatanova J. (2009). Robust methods for purification of histones from cultured mammalian cells with the preservation of their native modifications. Nucleic Acids Res. 37:e81. 10.1093/nar/gkp273 19443446 PMC2699528

[B65] Rodríguez-Durán L. F. Castillo D. V. Moguel-González M. Escobar M. L. (2011). Conditioned taste aversion modifies persistently the subsequent induction of neocortical long-term potentiation in vivo. Neurobiol. Learn. Mem. 95, 519–526. 10.1016/j.nlm.2011.03.003 21440652

[B66] Roozendaal B. Castello N. A. Vedana G. Barsegyan A. McGaugh J. L. (2008). Noradrenergic activation of the basolateral amygdala modulates consolidation of object recognition memory. Neurobiol. Learn. Mem. 90, 576–579. 10.1016/j.nlm.2008.06.010 18657626 PMC2572617

[B67] Roozendaal B. Hernandez A. Cabrera S. M. Hagewoud R. Malvaez M. Stefanko D. P.. (2010). Membrane-associated glucocorticoid activity is necessary for modulation of long-term memory via chromatin modification. J. Neurosci. 30, 5037–5046. 10.1523/JNEUROSCI.5717-09.2010 20371824 PMC2861482

[B68] Roozendaal B. McEwen B. S. Chattarji S. (2009). Stress, memory and the amygdala. Nat. Rev. Neurosci. 10, 423–433. 10.1038/nrn2651 19469026

[B69] Roozendaal B. McGaugh J. L. (1996). Amygdaloid nuclei lesions differentially affect glucocorticoid-induced memory enhancement in an inhibitory avoidance task. Neurobiol. Learn. Mem. 65, 1–8. 10.1006/nlme.1996.0001 8673403

[B70] Roozendaal B. McGaugh J. L. (1997). Glucocorticoid receptor agonist and antagonist administration into the basolateral but not central amygdala modulates memory storage. Neurobiol. Learn. Mem. 67, 176–179. 10.1006/nlme.1996.3765 9075247

[B71] Roozendaal B. McGaugh J. L. (2011). Memory modulation. Behav. Neurosci. 125, 797–824. 10.1037/a0026187 22122145 PMC3236701

[B72] Roozendaal B. Okuda S. Van der Zee E. A. McGaugh J. L. (2006). Glucocorticoid enhancement of memory requires arousal-induced noradrenergic activation in the basolateral amygdala. Proc. Natl. Acad. Sci. U S A 103, 6741–6746. 10.1073/pnas.0601874103 16611726 PMC1458951

[B73] Roozendaal B. Quirarte G. L. McGaugh J. L. (2002). Glucocorticoids interact with the basolateral amygdala beta-adrenoceptor-cAMP/PKA system in influencing memory consolidation. Eur. J. Neurosci. 15, 553–560. 10.1046/j.0953-816x.2001.01876.x 11876783

[B74] Rumbaugh G. Miller C. A. (2011). Epigenetic changes in the brain: measuring global histone modifications. Methods Mol. Biol. 670, 263–274. 10.1007/978-1-60761-744-0_18 20967596 PMC3235043

[B75] Shema R. Sacktor T. C. Dudai Y. (2007). Rapid erasure of long-term memory associations in the cortex by an inhibitor of PKM zeta. Science 317, 951–953. 10.1126/science.1144334 17702943

[B76] Shi C. J. Cassell M. D. (1998). Cortical, thalamic and amygdaloid connections of the anterior and posterior insular cortices. J. Comp. Neurol. 399, 440–468. 10.1002/(SICI)1096-9861(19981005)399:4<440::AID-CNE2>3.0.co;2-1 9741477

[B81] Stefanko D. P. Barrett R. M. Ly A. R. Reolon G. K. Wood M. A. (2009). Modulation of long-term memory for object recognition via HDAC inhibition. Proc. Natl. Acad. Sci. U S A 106, 9447–9452. 10.1073/pnas.0903964106 19470462 PMC2695069

[B77] Stehberg J. Moraga-Amaro R. Simon F. (2011). The role of the insular cortex in taste function. Neurobiol. Learn. Mem. 96, 130–135. 10.1016/j.nlm.2011.03.005 21447397

[B78] Swank M. W. Sweatt J. D. (2001). Increased histone acetyltransferase and lysine acetyltransferase activity and biphasic activation of the ERK/RSK cascade in insular cortex during novel taste learning. J. Neurosci. 21, 3383–3391. 11331368 10.1523/JNEUROSCI.21-10-03383.2001 PMC6762472

[B79] van Stegeren A. H. Roozendaal B. Kindt M. Wolf O. T. Joëls M. (2010). Interacting noradrenergic and corticosteroid systems shift human brain activation patterns during encoding. Neurobiol. Learn. Mem. 93, 56–65. 10.1016/j.nlm.2009.08.004 19695335

[B80] Vecsey C. Hawk J. D. Lattal K. M. Stein J. M. Fabian S. A. Attner M. A.. (2007). Histone deacetylase inhibitors enhance memory and synaptic plasticity via CREB:CBP-dependent transcriptional activation. J. Neurosci. 27, 6128–6140. 10.1523/jneurosci.0296-07.2007 17553985 PMC2925045

